# Identification of gene isoforms and their switching events between male and female embryos of the parthenogenetic crustacean *Daphnia magna*

**DOI:** 10.1038/s41598-024-59774-1

**Published:** 2024-04-30

**Authors:** Yasuhiko Kato, Joel H. Nitta, Christelle Alexa Garcia Perez, Nikko Adhitama, Pijar Religia, Atsushi Toyoda, Wataru Iwasaki, Hajime Watanabe

**Affiliations:** 1 Department of Biotechnology, Graduate School of Engineering, Osaka University, Suita, Osaka Japan; 2 Institute for Open and Transdisciplinary Research Initiatives (OTRI), Osaka University, Suita, Osaka Japan; 3 Department of Integrated Biosciences, Graduate School of Frontier Sciences, The University of Tokyo, Tokyo, Japan; 4 Advanced Genomics Center, National Institute of Genetics, Mishima, Shizuoka Japan

**Keywords:** Gene expression, Evolutionary developmental biology

## Abstract

The cladoceran crustacean *Daphnia* exhibits phenotypic plasticity, a phenomenon that leads to diverse phenotypes from one genome. Alternative usage of gene isoforms has been considered a key gene regulation mechanism for controlling different phenotypes. However, to understand the phenotypic plasticity of *Daphnia*, gene isoforms have not been comprehensively analyzed. Here we identified 25,654 transcripts derived from the 9710 genes expressed during environmental sex determination of *Daphnia magna* using the long-read RNA-Seq with PacBio Iso-Seq. We found that 14,924 transcripts were previously unidentified and 5713 genes produced two or more isoforms. By a combination of Illumina short-read RNA-Seq, we detected 824 genes that implemented switching of the highest expressed isoform between females and males. Among the 824 genes, we found isoform switching of an ortholog of CREB-regulated transcription coactivator, a major regulator of carbohydrate metabolism in animals, and a correlation of this switching event with the sexually dimorphic expression of carbohydrate metabolic genes. These results suggest that a comprehensive catalog of isoforms may lead to understanding the molecular basis for environmental sex determination of *Daphnia*. We also infer the applicability of the full-length isoform analyses to the elucidation of phenotypic plasticity in *Daphnia*.

## Introduction

The cladoceran crustacean *Daphnia* is a zooplankton that lives widely in freshwater and brackish water^1^. This zooplankton group reproduces with facultative parthenogenesis that switches between clonal and sexual reproduction^2^. The genetically identical clones exhibit diverse phenotypes to adapt to environmental changes. The presence of predators leads to the development of unique structures such as helmets^3^ and neck teeth^4^. Hypoxia increases hemoglobin levels in the hemolymph, which changes the body color from pale to red^5^. In addition, the same genotype produces different types of sex, females and males. In a healthy population, *Daphnia* produces only females by parthenogenesis whereas a shortened photoperiod, a lack of food, and/or increased population density, stimulate the parthenogenetic production of males that involves sexual reproduction for producing resting eggs^2^. To understand the environmental adaptation of *Daphnia* at a genome level, the complete genome has been reported in several *Daphnia* species including *Daphnia pulex*^6,7^, *Daphnia longispina*^8^, *Daphnia sinensis*^9^, and *Daphnia magna*^10–12^. To investigate the function of the identified genes, genetic engineering tools for loss-of- and gain-of-function analyses have been established mostly in *D. magna*^13–19^, which enables us to do genetic research in this species.

Among the phenotypic plasticity of *D. magna*, the environmental sex determination has been the most investigated at a molecular level^20,21^. In *Daphnia* and the other cladoceran crustaceans, parthenogenetic females detect environmental changes and commit the oocytes to be developed into males by sesquiterpenoid signaling^22–24^. During the development of the offspring that is sexually committed to males, the DM domain-transcription factor Doublesex1 (Dsx1) is expressed and leads to the determination and maintenance of male characteristics^25,26^. We previously analyzed differential gene expression (DGE) between males, females, and Dsx1 mutants using short-read RNA-Seq, which revealed sex-biased genes and their correlation with Dsx1 gene activity^27^.

In eukaryotes, multiple isoforms are expressed from a single gene locus by post-transcriptional regulation such as exon-skipping and intron retention. This gene regulation leads to a variety of protein isoforms with different activity and localization, which finally results in different phenotypes such as development patterns including sexual dimorphism^28^. For instance, in insects, the generation of sex-specific isoforms of regulatory factors such as Transformer and Doublesex is responsible for sex determination^29^. Alternative usage of gene isoforms is also considered a key regulatory mechanism for controlling phenotypic plasticity^30,31^. However, in *D. magna*, the dimorphic expression of gene isoforms has not been analyzed yet due to the limitation of the short-read RNA-Seq^32^.

The long-read RNA-Seq methods using PacBio Iso-Seq or Nanopore sequencing have been used to identify the gene isoforms. Each isoform expression level can be analyzed by a combination of the long-read and short-read RNA-Seq^33,34^. The cumulative abundance of all isoforms from each gene indicates the expression level of the individual gene, which is applied for the DGE analysis^34,35^. Differential expression analysis of each isoform leads to the identification of genes showing differential isoform usage (DIU), which results in the detection of isoform-switching events between different conditions^34^. In this study, we aimed to catalog the mRNA isoforms and examine the sexual dimorphism of their expressions in *D. magna*. We used PacBio Iso-Seq for the identification of the full-length transcripts and Illumina short-read RNA-Seq for the quantitation of each isoform expression.

## Results and discussion

### Transcriptomes of *D. magna* male and female embryos were sequenced with PacBio Iso-seq and Illumina Hiseq

**Figure 1:**
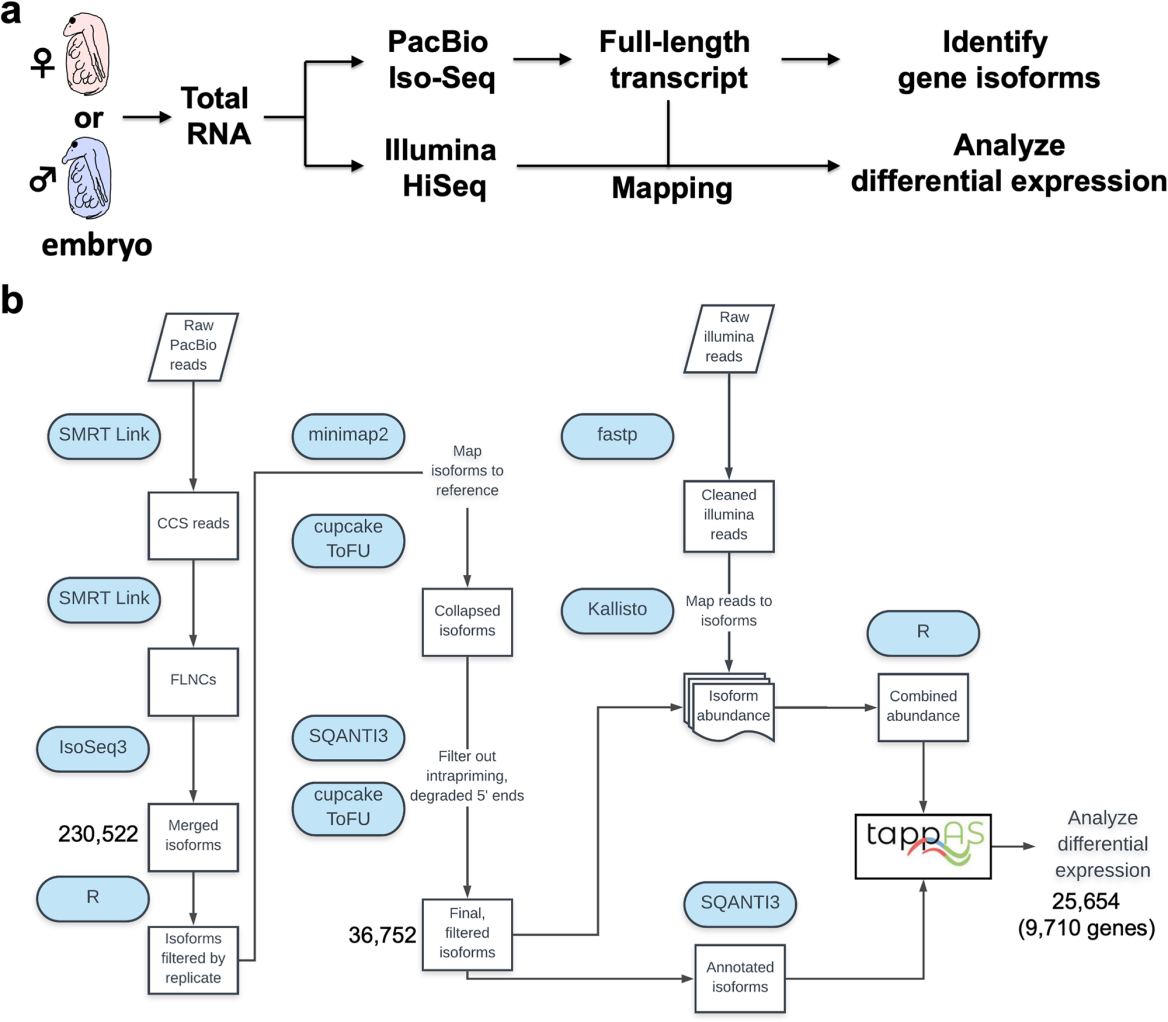
*Daphnia* embryo transcriptome workflow. (**a**) Overview of analysis in this study. Total RNAs were prepared from female or male embryos at 40 h after ovulation and divided into two samples each of which is subjected to PacBio Iso-Seq or Illumina HiSeq. Data of full-length transcripts from Iso-seq analysis not only led to the identification of gene isoforms but also was used for mapping the reads from Hiseq to analyze differential gene expression. (**b**) Workflow up to differential expression analysis in tappAS. CCS, circular consensus reads; FLNCs, full-length non-concatemers. The numbers of merged isoforms and final filtered isoforms were shown in addition to the numbers of isoforms and genes subjected to differential expression analysis.

To investigate sexually dimorphic isoforms in genetically identical males and females of *D. magna*, we collected male and female embryos separately at 40 h after ovulation (Fig. 1a). This timing approximately corresponds to the embryonic stage 11 at which carapace partially covers the legs and sex difference of morphology of the first antennae is observed^36^. At the same stage, the master transcription factor Dsx1 for male determination has been already expressed^25,26^ and sex-differential transcriptome was found by Illumina short-read sequencing^27^. These previous results prompted us to choose this stage for the isoform-level transcriptome analysis in this study. Transcriptomes of six samples (three males’ and three females’ samples) of *D. magna* were obtained using PacBio Iso-Seq and Illumina HiSeq.

### Identification of 25,654 transcripts from 9710 genes

We first merged all the long-read Iso-Seq data across samples and replicates, resulting in a set of 230,522 high-quality isoforms. Those isoforms were filtered and mapped to a reference genome (GenBank accession no. GCF_020631705.1) as detailed in Fig. 1b, which led to 36,752 filtered and non-redundant isoforms. To evaluate the sexually dimorphic expression of each isoform, we mapped the short-read RNA-seq data of males or females to the final set of 36,752 isoforms (Fig. 1a, analysis of differential expression). During this process, we eliminated transcripts with low counts by using the tappAS software and finally obtained 25,654 transcripts from 9710 genes (Fig. 1b, Supplementary Table S1, Supplementary Table S2).

### Diverse and multiple isoforms from known and novel genes

**Figure 2:**
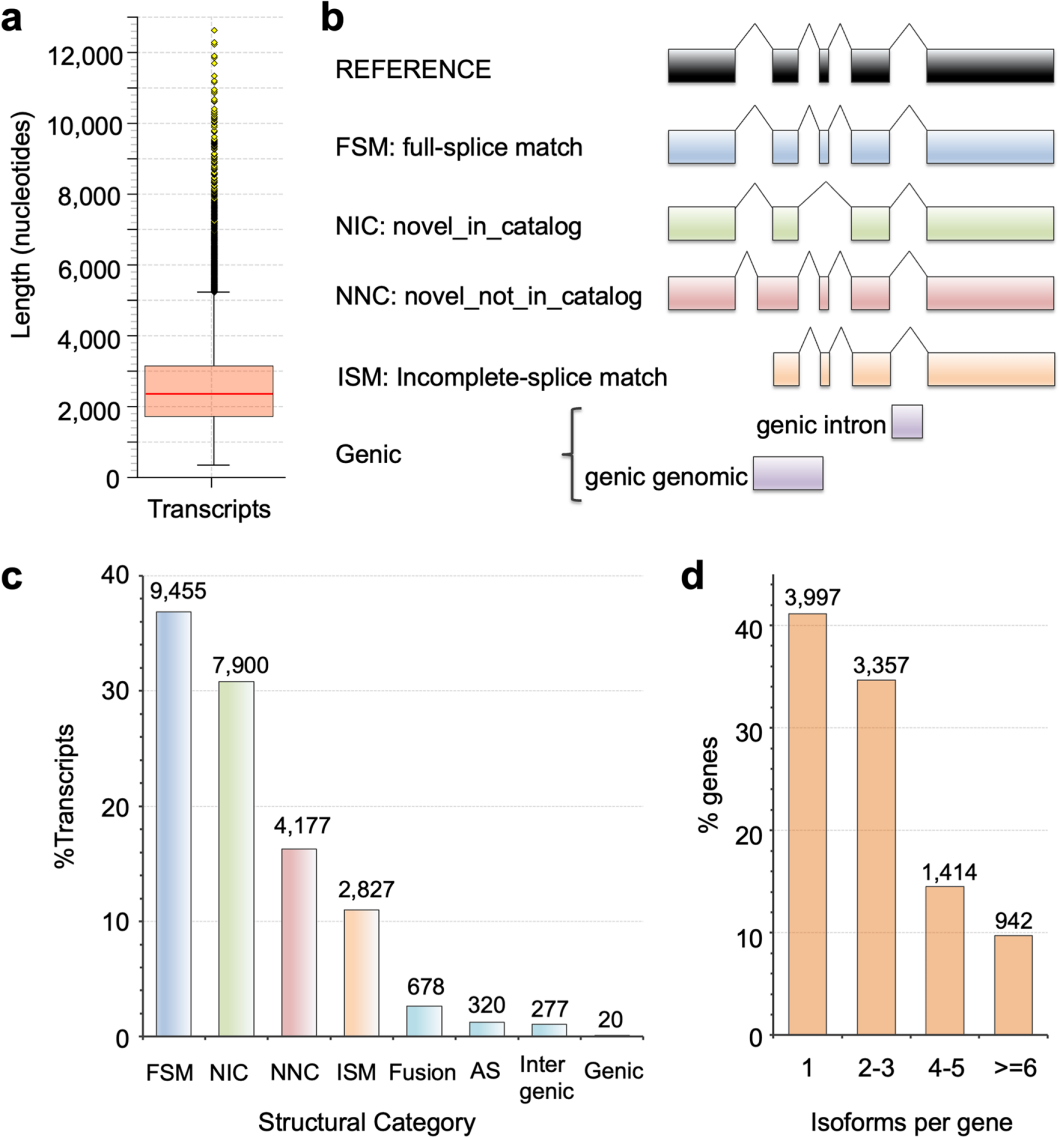
Characterization of gene isoforms. (**a**) Transcript lengths. The length distribution was displayed in nucleotides for all transcripts. (**b**) Classification of isoforms with SQANTI3. Each isoform structure was compared with the reference transcriptome. FSM, transcript spliced the same as a reference transcript at all splice junctions. NIC, novel transcript using already annotated splice junctions or novel splice junctions formed from already annotated donors and acceptors. NNC, novel transcript using novel donors and /or acceptors. ISM, novel transcript that uses already annotated splice junctions but does not match a reference transcript at all splice junctions. Genic intron, transcript located within an annotated intron. Genic genomic, transcript that overlaps with a part of annotated exons and intron/intergenic regions. (**c**) % transcripts categorized into Fusion, AS (antisense), Intergenic, and the other five structures shown in (**b**). Fusion, transcript spanning two annotated genes. AS, transcript overlapping complementarily with an annotated transcript. Intergenic, transcript located outside of an annotated gene. The number of transcripts in each category was shown above each bar. (**d**) Isoforms per gene. The distribution of isoforms per gene was displayed.

The length of the 25,654 transcripts ranged from 0.316 to 12.6 kilobases (Fig. 2a). Of those transcripts, 95.0% (24,379 transcripts) originated from the previously annotated 8872 genes. In detail, 36.9% (9455 transcripts) showed perfect matches to a reference transcript (FSM, Fig. 2b, c) whereas 58.2% (14,924 transcripts) were categorized as previously unidentified isoforms (NIC, 30.8%; NNC, 16.3%; ISM, 11.0%; genic, 0.0780%) (Fig. 2b, c). The remaining 4.97% (1275 transcripts) was derived from 838 novel genes including 277 intergenic, 177 antisense, and 384 fusion genes (Fig. 2b, c, Supplementary Table S1). We also counted the number of isoforms per gene and found that 5713 genes (58.8%) produced two or more isoforms (Fig. 2d, Supplementary Table S2).

### Isoform switching events occur between genetically identical males and females

We performed differential expression analyses with multiple isoforms-producing genes whose number was reduced from 5713 to 4638 by filtering genes with the less abundant isoforms (Supplementary Table S3). First, to examine differential gene expression (DGE), we calculated the cumulative abundance of all isoforms from each gene and compared it between males and females. Among the 4,638 genes, 615 showed significance for DGE (Table 1). Second, we analyzed differential expression at an isoform level and identified 122 genes showing differential isoform usage (DIU genes). Of the DIU genes, 77.9% (95 genes) were grouped into genes that did not show DGE (Table 1). Third, we examined how many genes implemented the switching of the highest expressed isoform (named major isoform switching^34^) between males and females. Of the 4638 multiple isoform-producing genes, the major isoform switching occurred in 824 genes, among which 87.7% (723 genes) were not categorized as DGE genes (Table 1) (Supplementary Table S3). These results suggest that not only DGE but also DIU and isoform switching may be important for the sexual dimorphism of *D. magna*.

**Table 1:** Number of genes showing differential gene expression (DGE), differential isoform usage (DIU), and major isoform switching between males and females.

	DIU	Not DIU	Total
DGE	27 (13)	588 (88)	615 (101)
Not DGE	95 (39)	3928 (684)	4023 (723)
Total	122 (52)	4516 (772)	4638 (824)

The number of genes showing major isoform switching was shown in the parenthesis.

### Regulators of biosynthetic and metabolic processes tend to be spliced in a sex-dependent manner

**Figure 3:**
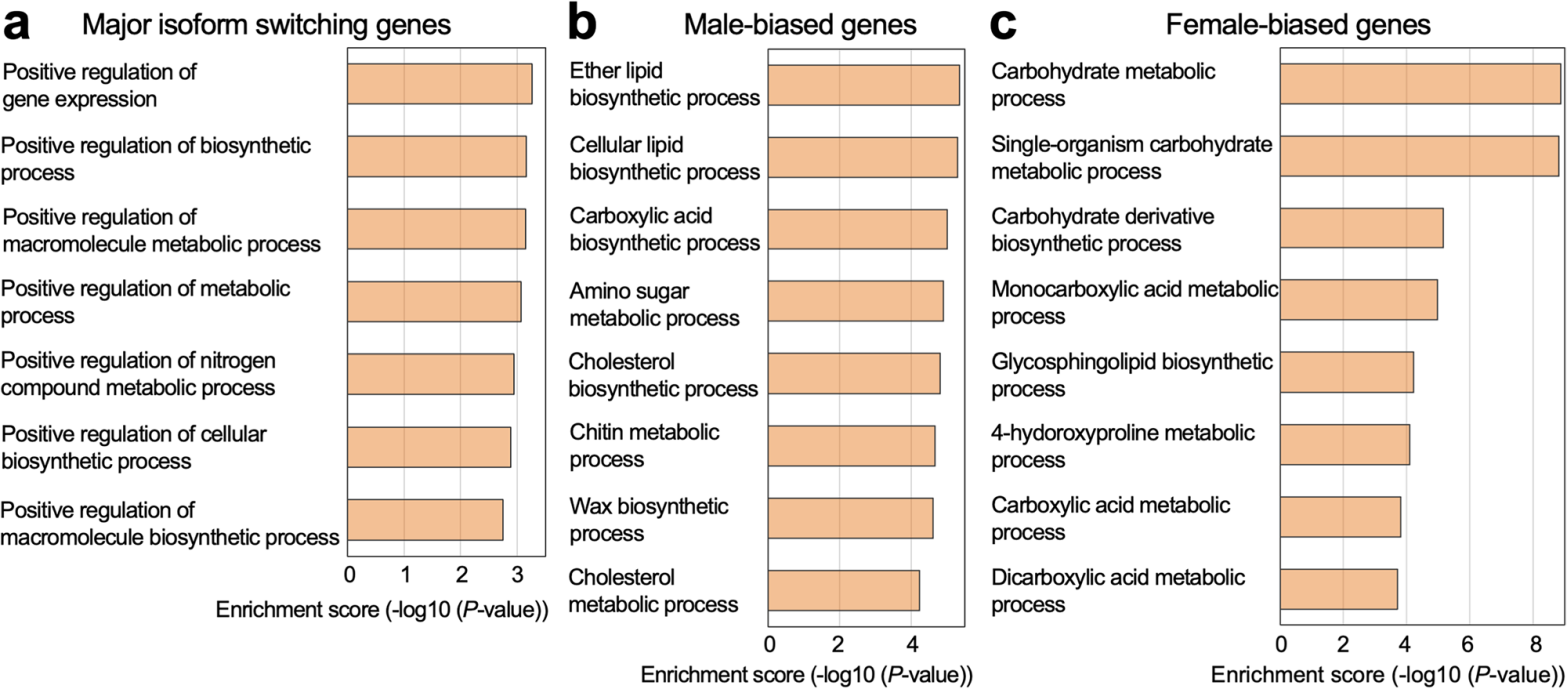
Gene ontology (GO) enrichment analysis. (**a**) GO (− log10 (*p* value)) terms of the genes showing major isoform switch. Biological processes related to the positive regulation of metabolic and biosynthetic processes were listed in the graph. All the multiple isoform-producing genes were used as the comparing list. (**b**) GO (− log10 (*p* value)) terms of the male-biased genes. Biological processes related to the metabolic and biosynthetic processes were listed in the graph. (**c**) GO (− log10 (*p* value)) terms of the female-biased genes. Biological processes related to the metabolic and biosynthetic processes were listed in the graph. In GO analyses of the male- and female-biased genes, all genes were used as the comparing list.

To investigate what kind of cellular process the isoform switching is involved in, we ran a gene ontology (GO) enrichment analysis of the 824 major isoform-switching genes using the “Biological Processes” categories. Among the 91 enriched categories, the top seven categories were related to the positive regulation of biosynthetic and metabolic processes (Fig. 3a, Supplementary Table S4), suggesting that expressions of biosynthetic and metabolic genes were different between males and females. To examine this possibility, we investigated “Biological Processes” categories enriched in DGE genes that were divided into two groups, male-biased and female-biased genes (Supplementary Table S5, Supplementary Table S6). Testing a list of the male-biased genes found enrichment of 157 GO categories, among which there were processes involved in the biosynthesis of lipids including cholesterol and wax in addition to carboxylic acid biosynthesis, amino sugar metabolism, chitin biosynthesis, and cholesterol metabolism processes (Fig. 3b, Supplementary Table S7). Using a list of the female-biased genes, we found 382 GO categories, among which terms associated with the carbohydrate metabolic process were more prominently enriched compared to the other terms related to biosynthesis and metabolism (Fig. 3c, Supplementary Table S8).

### Isoform switching of an ortholog of CRTC, a major regulator of carbohydrate metabolism

To assume a potential mechanism for the upregulation of genes related to the carbohydrate metabolic process in females, we examined if carbohydrate metabolism regulators were included in the major isoform switching genes (Supplementary Tables S9–S15). We found the isoform switching event of an ortholog of CREB-regulated transcription coactivator (CRTC) that controls and appears to regulate glucose metabolism in humans^37^ and *Drosophila*^38^ respectively. In mammals, the gluconeogenesis regulator CRTC2 protein is phosphorylated in the conserved serine-containing regions at the basal state and interacts with 14–3–3 protein, resulting in the sequestration of the CRTC2 in the cytoplasm. In response to cellular stimuli, the CRTC2 protein is dephosphorylated, moved into the nucleus, and associated with the transcription factor CREB, leading to the activation of the target genes^39^.

**Figure 4:**
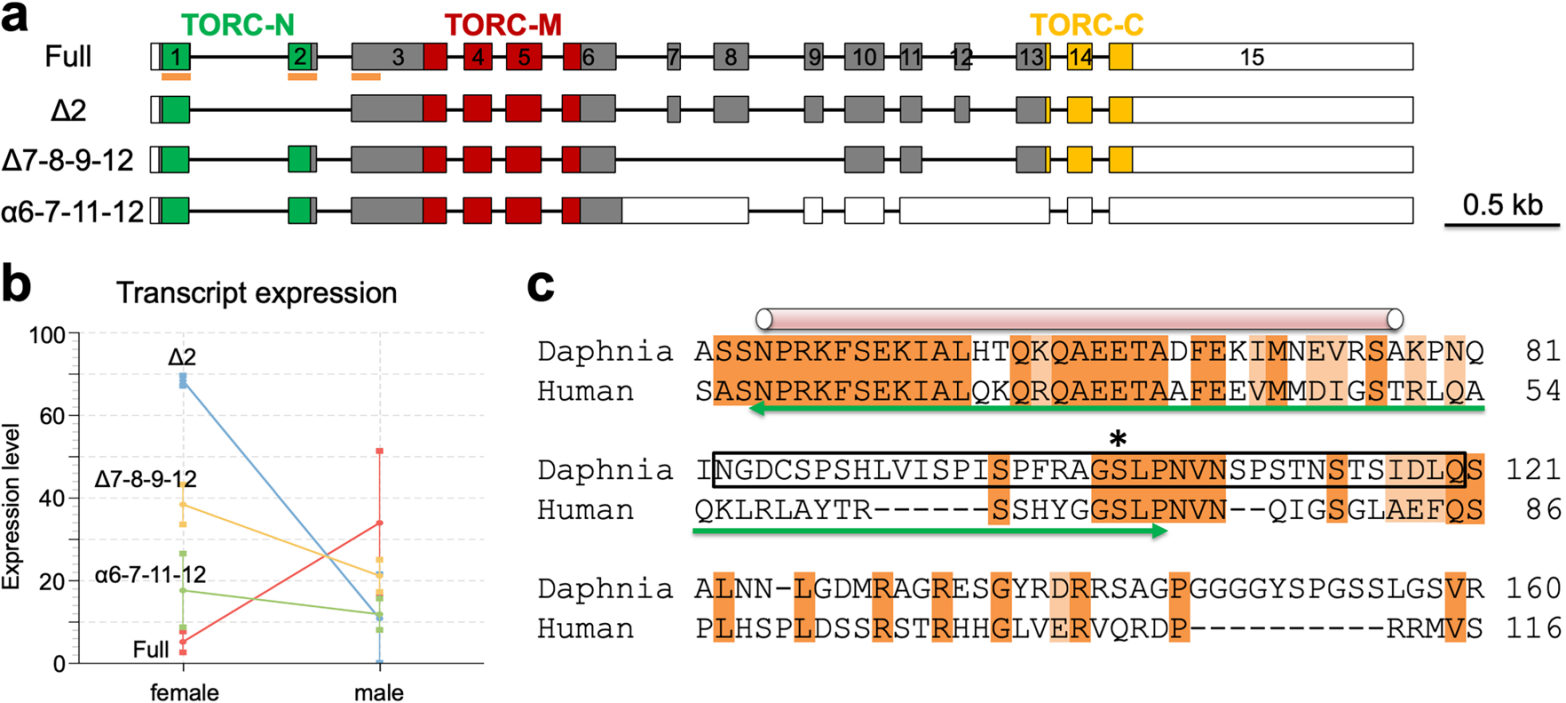
Sexually dimorphic expression of the CRTC gene in *D. magna*. (**a**) Introns and exons of four different isoforms of the CRTC gene. Colored and white boxes indicate coding and untranslated regions respectively. In the coding sequence, green, red, and yellow, regions show TORC-N, TORC-M, and TORC-C domain-coding regions. The remaining coding regions were colored with grey. The orange bars under the structure of the full isoform indicate the coding region used for amino acid alignment in (**c**). (**b**) Comparison of the expression level of each isoform between males and females. (**c**) Amino acid sequence alignment of the TORC-N domain and its surrounding region of human CRTC2 and *D. magna* CRTC. The corresponding region was shown with the orange bars in (**a**). The TORC-N domain was indicated with the green color of the two-headed arrow. In the *D. magna* CRTC gene, the exon 2 codes for the amino acid sequence enclosed in a square. The cylinder indicates a continuous alpha-helix of the CREB-binding domain of human CRTC2. The asterisk indicates a potential phosphorylation site that is recognized by the 14–3–3 protein. Identical residues are highlighted in orange and residues with similar characteristics in light orange.

The long read Iso-Seq identified four isoforms of the *Daphnia* CRTC gene, full, Δ2, Δ7–8–9–12, and α6–7–11–12 (Fig. 4a). Of the four, sex-dependent isoform switching occurred between the full and Δ2 isoforms. In males, the full and Δ2 isoforms were the most and least abundant respectively whereas the reciprocal expression pattern was observed in females (Fig. 4b). The full isoform codes for three domains conserved in the CRTC family, TORC-N, TORC-M, and TORC-C (Fig. 4a). Importantly, the Δ2 isoform lacks exon 2, leading to the truncation of the TORC-N domain (Fig. 4a, c, green lines with arrows). This domain contains a continuous alpha-helix for binding to CREB (Fig. 4c, cylinder). It also includes three amino acid residues required for association with 14–3–3 protein, Ser103 as a potential phosphorylation site (Fig. 4c, asterisk) and the following Leu104 and Pro105 for enhancing the hydrophobic interaction between CRTC and 14–3–3 protein (Fig. 4c)^40^. The exon 2 skipping in the Δ2 isoform resulted in the exclusion of the 14–3–3 interaction motif (S103-L104-P105). Because the mutation of the 14–3–3 interacting residue of the CRTC has been reported to enhance activation of the target gene^41^, the product of the Δ2 isoform may have higher transactivation ability, which in turn is likely to be involved in upregulation of carbohydrate metabolic genes in females.

## Conclusion

In this study, we identified gene isoforms and their switching events between clonal males and females, which is potentially important for environmental sex determination and sexual dimorphism in *D. magna*. The function of the sex-specific isoforms could be analyzed by targeting sex-specific exon and exon/intron junction sequences using the gene manipulation tools in this species. We anticipate that the approach and resources of this study contribute to understanding environmental sex determination mechanisms in *D. magna*. We also infer the great potential of the full-length transcript analysis for studying phenotypic plasticity in *Daphnia* species.

## Materials and methods

### *Daphnia magna* stain and culture condition

The *Daphnia magna* strain (NIES clone) was obtained from the National Institute for Environmental Studies (NIES; Tsukuba, Japan) and was maintained at 23 ± 1 °C with a density of 16 individuals/L under a 16 h light/8 h dark photoperiod in 5 L of Aachener Daphnien Medium (ADaM) medium^42^. Eighty juveniles (less than 24 h old) were collected, cultured, and fed daily with 8 × 10^9^ cells of *Chlorella vulgaris* (Oita Medaka Biyori, Oita, Japan) and 3 mg of baker’s yeast (Marusan Pantry, Ehime, Japan) during the first week. Later, juveniles were removed daily and amounts of chlorella and yeast extract were doubled. The culture medium was changed once a week. For inducing male production, adults of female *D. magna* (2–3-weeks old) were exposed to 1 µg/L of the synthetic juvenile hormone analog, Fenoxycarb (FUJI FILM Wako Pure Chemical cooperation, Osaka, Japan) ^26^ for 16 h during the critical period of sex determination^24,43^. After ovulation, the exposed adults were transferred to Fenoxycarb-free ADaM medium, cultured for 40 h, and dissected for collecting male embryos.

### Total RNA preparation

Female or male embryos at 40 h after ovulation were used. Total RNAs were extracted using the RNeasy Plus Micro Kit (Qiagen, Valencia, CA, USA), purified with phenol/chloroform/isoamyl alcohol (25:24:1, v/v/v, pH5.2), precipitated with Ethanol, and dissolved in DNase/RNase-free distilled water (Life Technologies Corporation, Grand Island, NY, USA). The integrity of total RNA was assessed on the Agilent 2100 Bioanalyzer (Agilent Technologies, Santa Clara, CA, USA).

### Iso-Seq library preparation and sequencing

The male and female samples from *D. magna* were prepared for long-read isoform sequencing (Iso-Seq). Three biological replicates for each sample were conducted. The full-length cDNA was produced from 300 ng of total RNA for each sample using the NEBNext Single Cell/Low Input cDNA Synthesis & Amplification Module (New England Biolabs, MA USA) and Iso-Seq Express Oligo kit (Pacific Biosciences, CA USA) according to manufacturer’s protocols. Additionally, ribosomal RNA (rRNA) was removed from 12 μg of total RNA of each sample using the Ribo-Zero Gold kit (Illumina, CA USA). The rRNA-depleted RNA samples were then polyadenylated using the SMARTer smRNA-Seq kit for Illumina (Clontech Laboratories, Inc., CA USA). The full-length cDNA was generated following the same protocols as those used for total RNA. After size selection with the ProNex beads (Promega, WI USA), the full-length cDNA libraries were constructed with the SMRTbell Express kit 2.0 (Pacific Biosciences, CA USA). The concentration and the size distribution of each library were measured using the Qubit 4 Fluorometer (Thermo Fisher Scientific, MA, USA) and Agilent 2100 Bioanalyzer (Agilent Technologies, CA, USA), respectively. Each library was then sequenced on the Sequel system (Pacific Biosciences, CA USA) using one SMRT Cell 1 M v3 LR with Binding Kit 3.0 and Sequencing Kit 3.0, with a collection time of 20 h.

### Illumina short-read library preparation and sequencing

Total RNA was extracted from the male and female samples from *D. magna*. Sequencing libraries were constructed using the TruSeq Stranded Total RNA Library Gold (Illumina, CA, USA). Three biological replicates for each sample were conducted. All these procedures were performed according to the manufacturer’s instructions. Single-end sequencing was conducted on the Illumina HiSeq 2500 system with 100 bp read length.

### Isoform filtering

PacBio SMRT Link v8 (Pacific Biosciences, Menlo Park, CA, USA) was used to convert raw PacBio reads (zero-mode waveguides) to circular consensus sequencing (CCS) reads, yielding 6,684,317 CCS reads total (mean 557,026 ± 28,183 reads per sample; all errors SD unless otherwise indicated). Following the removal of concatemers, 5,519,340 (82.6%) of the CCS reads were identified by SMRT Link as full-length non-concatemer transcripts (FLNCs) based on the presence of sequencing primers and poly-A tails, which were subsequently trimmed. FLNCs from all SMRT cells were then merged and clustered using IsoSeq v3 (https://github.com/PacificBiosciences/IsoSeq), resulting in a set of 230,522 high-quality isoforms. To account for sequencing artifacts, the high-quality isoforms were filtered to retain only those present in at least two of three replicates within a given treatment (e.g., at least 2/3 replicates of male treated with ribo-zero) using a custom R script in R v4.0.2^44^, resulting in 129,159 filtered, high-quality isoforms (56.0%). The filtered, high-quality isoforms were then mapped to a reference genome (*D. magna* NIES strain, GenBank accession no. GCF_020631705.1), using minimap2^45^. collapse_isoforms_by_sam.py in Cupcake ToFU (https://github.com/Magdoll/cDNA_Cupcake) was used to collapse isoforms mapping to identical genomic positions, resulting in 82,732 non-redundant isoforms (64.1%). filter_away_subset.py in Cupcake ToFU was used to filter away isoforms with degraded 5’ ends (artifacts that do not reflect real isoform variation), retaining 61,472 non-redundant isoforms (74.3%). sqanti3_RulesFilter.py in SQANTI3^46^ was used to filter away 23,748 (38.6%) isoforms with intrapriming sites (priming at genomic poly-A sites), non-canonical splicing, or RT switching, resulting in 36,752 filtered, non-redundant isoforms.

### Isoform annotation

Sqanti3_qc.py in SQANTI3 was run on the final isoforms to produce an annotated alignment file in GFF3 format compatible with tappAS^34^ using the –isoAnnotLite flag. The SQANTI3 annotations include among other information, characterization of structural category, location of open reading frames (ORFs), and categorization of genes, exons, and CDS^46^. EnTAP^47^ was used to annotate gene ontology (GO terms).

### Isoform quantification

Raw Illumina reads were cleaned using fastp^48^ on default settings, which automatically detects and removes adapter sequences and trims low-quality bases, resulting in 68,627,563 clean reads total (99.54% of original reads). kallisto^49^ was used to map the cleaned Illumina reads to the final, filtered isoforms, generating one TSV file with isoform abundances per sample. The TSV files were merged into a single file and formatted to be compatible with tappAS using a custom R script.

### GO enrichment analysis

GO enrichment analyses using a list of major isoform-switching genes and sex-biased genes (male-biased or female-biased) were conducted with the GOseq^50^ R package installed in the tappAS. Over-representation analysis with the Wallenius test was done using the “Biological Process” GO categories. A background gene list comprised of all multi-isoform genes and all genes was used for analyses using a list of major isoform-switching genes and sex-biased genes, respectively. GO categories with *p* values < 0.05 were considered significant.

### Supplementary Information

Supplementary Information.

## Data Availability

The data have been deposited with links to BioProject Accession Number PRJDB13788 in the DDBJ BioProject database.
